# Enhanced Electrochemical Properties of Catalyst by Phosphorous Addition for Direct Urea Fuel Cell

**DOI:** 10.3389/fchem.2020.00777

**Published:** 2020-10-19

**Authors:** Unho Lee, You Na Lee, Young Soo Yoon

**Affiliations:** Materials Science and Engineering, Gachon University, Seongnam-si, South Korea

**Keywords:** direct urea fuel cell, urea, phosphorous addition, anode catalyst, anion exchange fuel cell, hydrothermal synthesis, Ni-Pd alloy, MWCNTs

## Abstract

An anode bimetallic catalyst comprising Ni-Pd alloy nanoparticles was loaded on acid-treated multi-walled carbon nanotubes (MWCNTs) for application in a direct urea fuel cell. The bimetallic catalyst and MWCNTs were synthesized by a hydrothermal method at 160°C for 5 h. To reduce the catalyst particle size, alkaline resistance, and facilitate their uniform distribution on the surface of the MWCNTs, phosphorus (P) was added to the Ni-Pd/MWCNT catalyst. The effects of P on the distribution and reduction in size of catalyst particles were investigated by Brunauer–Emmett–Teller analysis, transmission electron microscopy, and X-ray diffraction analysis. The enhanced catalytic activity and durability of the P-containing catalyst was confirmed by the high current density [1897.76 mA/cm^2^ (vs. Ag/AgCl)] obtained at 0.45 V in a 3 M KOH/1.0 M urea alkaline aqueous solution compared with that of the catalyst without P [604.87 mA/cm^2^ (vs. Ag/AgCl)], as determined by cyclic voltammetry and chronoamperometry. A Urea–O_2_ fuel cell assembled with a membrane electrode assembly comprising the Ni-Pd(P)/MWCNT catalyst delivered peak power densities of 0.756 and 3.825 mW/cm^2^ at 25 and 60°C, respectively, in a 3 M KOH/1 M urea solution.

## Introduction

Fuel cells are well-known eco-friendly energy production devices that have several advantages (Ryu et al., 2014; Yoon et al., 2014, 2017) such as a high energy-conversion efficiency (Lim et al., 2014; Chen et al., 2017; Yu et al., 2018) that is 70% higher than that of fossil fuel-based energy production systems (Smalley, 2003; Xiao et al., 2015). In addition, fuel cells generate non-polluting gases such as H_2_O or CO_2_ as a byproduct and have a tremendous cost advantage over fossil fuel-based systems. Among the various fuel cells, direct urea fuel cells (DUFCs) can achieve high power densities because of the high theoretical energy density of urea (16.9 MJ L^−1^) that is used as a liquid fuel with high solubility (1,079 g L^−1^ at 20°C) (Ding et al., 2014, 2015; Liu et al., 2017). According to Equation 1, the overall reaction voltage of urea electrocatalytic oxidation (1.146 V) is similar to the open circuit voltage (OCV) of a hydrogen fuel cell (1.23 V). Thus, DUFCs are actively researched as next-generation fuel cells that can be a viable alternative to proton exchange membrane fuel cells. Moreover, the use of urea in fuel cells has some advantages. One of the technical advantages is that urea, a nitride-based compound that is widely used as a fertilizer, can be used as a portable and independent power source as it is non-flammable and non-explosive (Ye et al., 2015). Second, the OH^−^ ion generated during urea oxidation adsorbs on the Ni catalyst and easily forms compounds such as Ni(OH)_2_ and NiOOH (Wang et al., 2012, 2014; Periyasamy et al., 2016). Finally, urea exhibits high catalytic oxidizability without the use of Pt, which is an expensive precious metal; in addition, no catalytic poising by the byproduct, CO, is observed for the fuel cell reaction mechanism based on OH^−^ anion migration (Kwon et al., 2012a,b; Kim et al., 2018). However, the use of single-component Ni catalyst in the operating conditions of urea fuel cells leads to continuous voltage drop and a lower power density than the achievable value. This phenomenon is called overpotential in alkaline aqueous solution fuel cells (Kim et al., 2015; Lee et al., 2017a,b), which is the potential difference between the thermodynamically determined reduction potential and the potential at which the reduction reaction actually occurs. Alkaline fuel cells mainly suffer from the above-mentioned problems with an overvoltage of more than 0.4 V. These problems can be overcome by alloying different metals such as Co, Cr, Pd, Ir, and Cu with Ni to form bimetallic catalysts. The alloying of metal with Ni affords bimetallic catalysts with different atom sizes, and the active area can be increased by decreasing the activation overvoltage of the metals to reduce adsorption-desorption between the catalyst and OH^−^ ion. Ni-M (M = Co, Cr, Pd, Ir, etc.) (Lan et al., 2010; Lan and Tao, 2011; Guo et al., 2016) bimetallic catalysts result in chemical catalytic reactions, high current densities, and uniform catalyst properties. Among the various metals, Pd is a well-known catalyst that can effectively catalyze urea oxidation by regulating the activation energy of Ni metal and urea or catalytic-fuel reaction and facilitates fuel adsorption-desorption. However, controlling the structure of Ni-Pd bimetallic alloy catalyst particles and their dispersion in alkaline aqueous solutions is difficult. When Ni metal is reduced, the cohesion strength of Ni has very stable energy; hence, when catalyst materials are synthesized on a catalyst support with bimetallic components such as Ni-Pd, the active area decreases (Lee et al., 2013a; Xu et al., 2014). Nano-sized Ni-based metal catalysts have a high surface area-to-volume ratio that results in fast catalytic reactions; however, condensation reduces the surface energy. To overcome these problems, highly efficient catalysts with a high catalytic area and improved properties with the catalyst particles distributed evenly over the catalyst support are required (Guo et al., 2015). Multi-walled carbon nanotubes (MWCNTs) with a large surface area were used for catalyst distribution, and phosphorus (P) was added to the Ni-Pd bimetallic catalyst. MWCNTs, a well-known 1-D carbon material, have a high mechanical strength and are stable in acidic and alkaline media (Lee et al., 2012, 2013b, 2014). Characteristic with very high stability is synthesized with catalyst using surface treatment. When synthesized over a large area of catalyst support, the heterotopic catalyst Ni-Pd is unstable in form control and has a cohesive characteristic. To solve this problem, P was added to prevent aggregation and ensure uniform distribution. P is known to reduce the size of metal nanoparticles (Lee et al., 2013a; Liang et al., 2016; Ke et al., 2020), thus facilitating morphological control and uniform particle distribution. The addition of P to Ni-Pd prevents the occurrence of overpotential for urea oxidation and increases the active area. The electrochemical and structural properties of the catalysts with and without P were compared. P addition improved the properties of the catalyst, facilitated uniform dispersion, and resulted in increased electrochemical activity (Pham and Yoon, 2020). The evenly dispersed catalyst exhibited a better catalytic activity toward the fuel and effectively oxidized urea in alkaline aqueous solutions. The Ni-Pd/MWCNT catalyst was used to construct a fuel cell as a prototype power generator with a high power density.


$$\begin{array}{l} Anode\;:\;{CO\left({NH}_{2}\right)}_{2}\;+\;{6OH}^{-}\;\to N_{2}+{CO}_{2}+5H_{2}O+6e^{-}\\ \;(E_{0}=\;-0.746V\;vs.\;SHE)\\ Cathode\;:\;O_{2}+2H_{2}O+4e^{-}\;\to 4{OH}^{-}\\ \;(E_{0}=0.401V\;vs.\;SHE)\\ Overall\;:\;2CO{\left({NH}_{2}\right)}_{2}+{3O}_{2}\;\to \;2N_{2}+{2CO}_{2}+{4H}_{2}O\\ \;(E_{0}=1.146V\;vs.\;SHE) \end{array}$$

## Materials and Methods

Nickel(II) chloride hexahydrate (NiCl_2_.6H_2_O), palladium(II) chloride (PdCl_2_), Isopropanol 70% in H_2_O (IPA), Sodium Hydroxide, Pellets (NaOH), and sodium hypophosphite (NaPO_2_H_2_.H_2_O) as precursors of Ni, Pd, and P, respectively, were purchased from Sigma-Aldrich (USA). MWCNTs were purchased from Eco-CNT, South Korea. All chemicals were used as received without further purification.

### Preparation of Surface-Functionalized MWCNTs by Acid Treatment

For acid pretreatment, MWCNTs were distributed and refluxed in 200 ml of 70% nitric acid at 120°C for 4 h. The resulting surface-functionalized MWCNTs were washed with distilled water and ethyl alcohol until the pH reached 7 and dried at 80°C. The pretreated surface-functionalized MWCNTs can be combined with the catalyst.

### Preparation of Ni-Pd(P)/MWCNT Catalyst Anode

**Figure 1:**
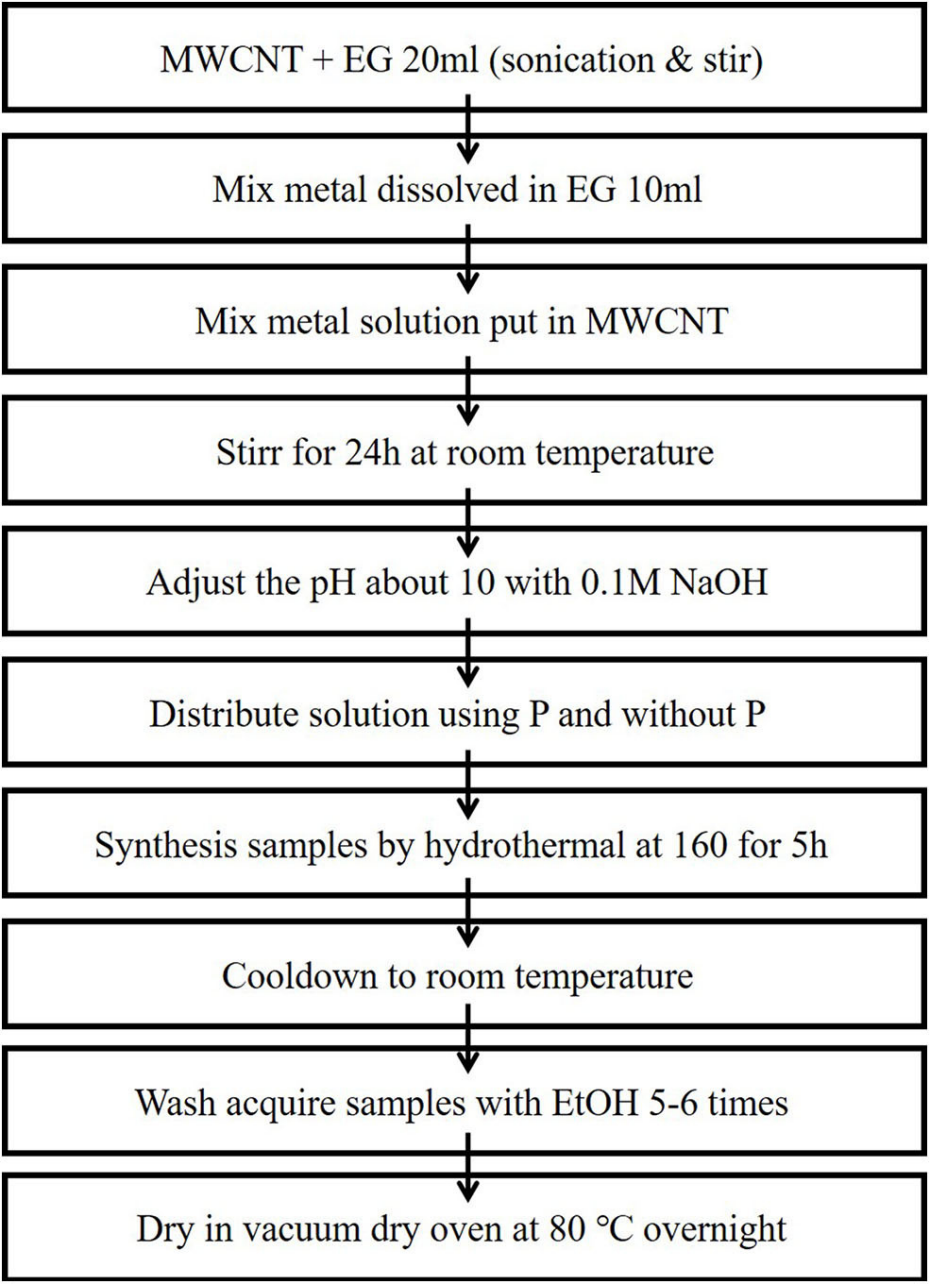
Synthesis of catalyst by hydrothermal method.

**Figure 2:**
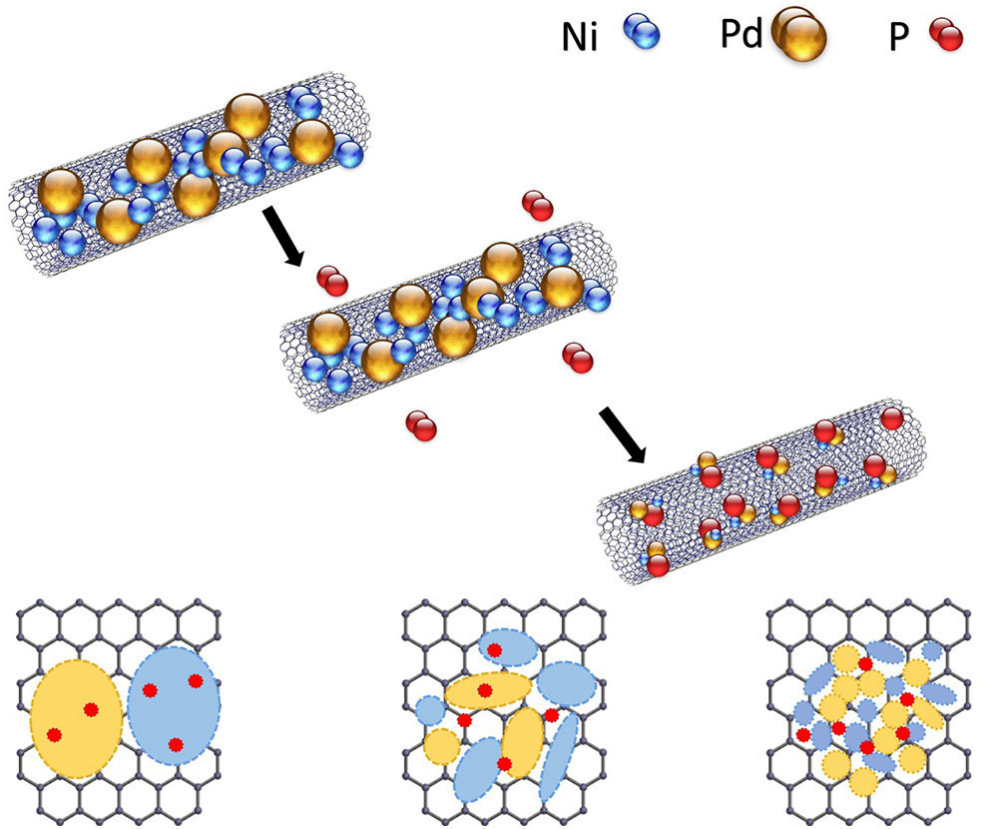
Schematic of reduction and size reduction of catalyst particles by P addition.

Ni-Pd bimetallic nanoparticles to be used as an anode were synthesized by a hydrothermal method (Figure 1) using surface-functionalized MWCNTs as the catalyst support. In a typical synthesis, 17.7 mg of PdCl_2_, 23.7 mg of NiCl_2_.6H_2_O, and 10.5 mg of NaPO_2_H_2_.H_2_O were mixed in a molar ratio of 1:1:1, and 40 mg of MWCNTs were added into 40 ml of ethylene glycol in a flask and stirred for an hour. In another flask, 40 mg of MWCNTs was dispersed in 25 ml of ethylene glycol by ultrasonication for 1 h (Ke et al., 2020). After the two mixtures were mixed and stirred for over 24 h, the pH was adjusted to ~10 with a 1 M NaOH solution. The mixtures were placed in an autoclave and heated at 160°C for 5 h. The obtained samples (Figure 2) were cooled to room temperature, washed with ethyl alcohol and acetone for five to six times, and dried in a vacuum oven at 80°C overnight.

### Characterization of Ni-Pd(P)/MWCNTs

The surface-functionalized and bimetallic catalyst-loaded MWCNTs were analyzed by transmission electron microscopy (TEM) (Tecnai G2 F30, FEI) for structural analysis of Ni-Pd(P) on the MWCNTs. The effects of P on the structural characteristics and shape control of the catalyst were determined by X-ray diffraction (XRD) (EMPyrean, Malvern Panalytical) analysis performed with Cu Kα (λ = 1.541Å) radiation with a fixed energy of 462 eV in the 2θ range of 10°–90°. The elemental composition and atomic ratios of Ni, Pd, and P of the catalyst on the catalyst support were determined by energy-dispersive X-ray spectroscopy (EDS) (ARM200-EDX, JEOL).

### Electrochemical Measurements

The electrochemical measurements of Ni-Pd/MWCNT and Ni-Pd(P)/MWCNT catalysts were performed in a three-electrode system using glassy carbon, Ag/AgCl, and a platinum wire as the working, reference, and counter electrodes, respectively. A catalyst slurry was prepared by ultrasonically dispersing 2.0 mg of the Ni-Pd(P)/MWCNT catalyst powder in 30 μl of Nafion solution 117 (5 wt%) and 200 μl of anhydrous isopropanol (99.9%). The catalyst slurry 3 μg was then loaded onto the surface of the glassy carbon working electrode. Cyclic voltammetry (CV) measurements were performed in the voltage window of 0–0.5 V at different scan rates ranging from 20 to 100 mV/s to determine the peak current density. The 0.33–1.0 M urea solution was used in a 1.0–3.0 M KOH solution for electrochemical analysis. The standard 3-electro-catalyst analysis for the measurement method were obtained based on the reference electrode, Ag/AgCl (sat. KCl). To determine the catalyst stability, chronoamperometry was performed to measure the variation in current with time using the working electrode composed of a 3 μm-thick catalyst slurry layer on a glassy carbon electrode. The solution was measured for CV test and after 3.0 M KOH/1.0 M urea solution electrochemical measure for 30,000 s. The catalytic-fuel characteristics were confirmed using the glassy carbon electrode and counter electrode. The reaction scan rate responded to the electrode and current density in a linear and constant increment. This result was used to measure the power density, and the propensity of the urea oxidation reaction was placed on the stabilization slope. For electrochemical analysis, CV and chronoamperometry were performed in a 3.0 M KOH/1.0 M urea solution.

### Fabrication of Unit Cell

A catalyst slurry composed of 40 mg of ink, 2.0 ml of isopropanol, and 0.6 ml of (5 wt%) Nafion solution was cast on a gas diffusion layer of 5 cm^2^ area. Then, 40 mg of catalyst was loaded by applying a 75 μm-thick layer by the doctor-blade method and dried at 70°C for over 12 h. It was heated and pressed with hot press machine that the anion exchange membrane, FAA-3-PK-130 membrane were heated and pressed with anode catalyst. Ni-Pd(P)/MWCNTs and cathode catalyst, Pt/C as catalyst layer on gas diffusion layer for the membrane electrode assembly (MEA) was pressed at 60°C for 5 min at 150 kg/cm^2^ by the hot press machine. After the MEA was loaded into the unit cell, the inlet location at the top of the bottom to gradually fill the fuel with the fuel as solution. The circuit is connected to determine the power density by controlling the voltage in the constant voltage mode. The area of the unit cell was 5 cm^2^, and a metal separator plate with an optimum flow path design for liquid fuel was used. The experiments were conducted at a normal OCV at 25 and 60°C, respectively. To ensure an optimal response of the catalyst, liquid fuel was filled from bottom to top, and this was done by setting the direction of the gas fuel cell and the fuel being injected. The power density was determined by measuring the OCV for 8 h until the voltage stabilized at 25 and 60°C. After the voltage stabilized, the constant voltage was reduced every 30 s in 0.05 V steps from the maximum OCV to the minimum voltage of 0.4 V. Thereafter, the loader was activated to determine the current density in the constant voltage mode. The repeated test was carried out current density to determine the optimum voltage. The unit cell performance of the fuel cell was measured in the constant current mode with 0.1 A increment at every 30 s at the OCV. The purpose of this measurement method was to determine the optimum current density and maximum power density. The fuel solution, 3.0 M KOH/1.0 M urea, was injected into the anode side at a rate of 5 ml/min, and 200 sccm of humidified air with oxygen was injected into the cathode side.

## Results

### Morphology and Structure of the Synthesized Catalyst

**Figure 3:**
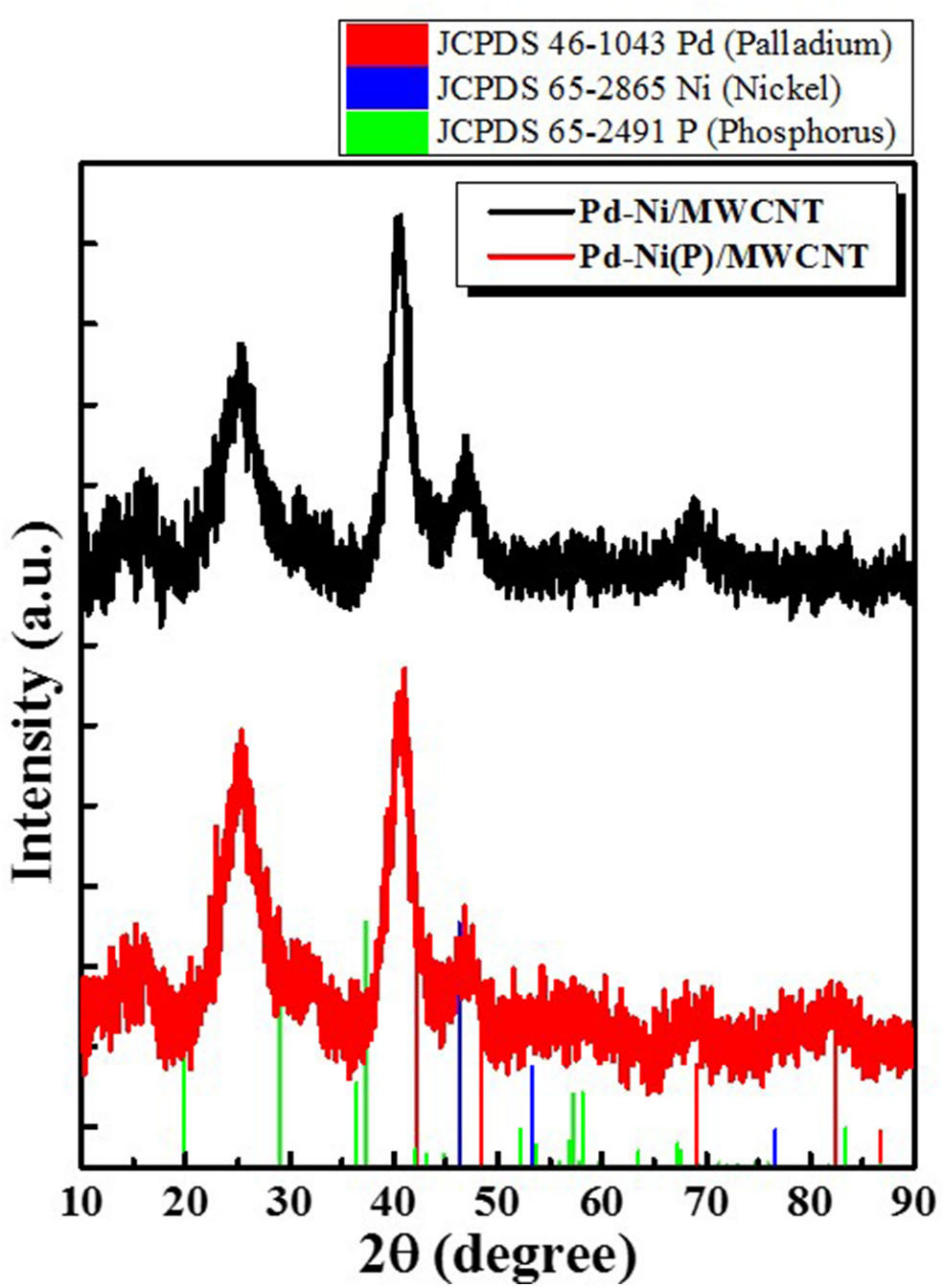
XRD profiles of Ni-Pd/MWCNT and Ni-Pd(P)/MWCNT catalysts.

**Figure 4:**
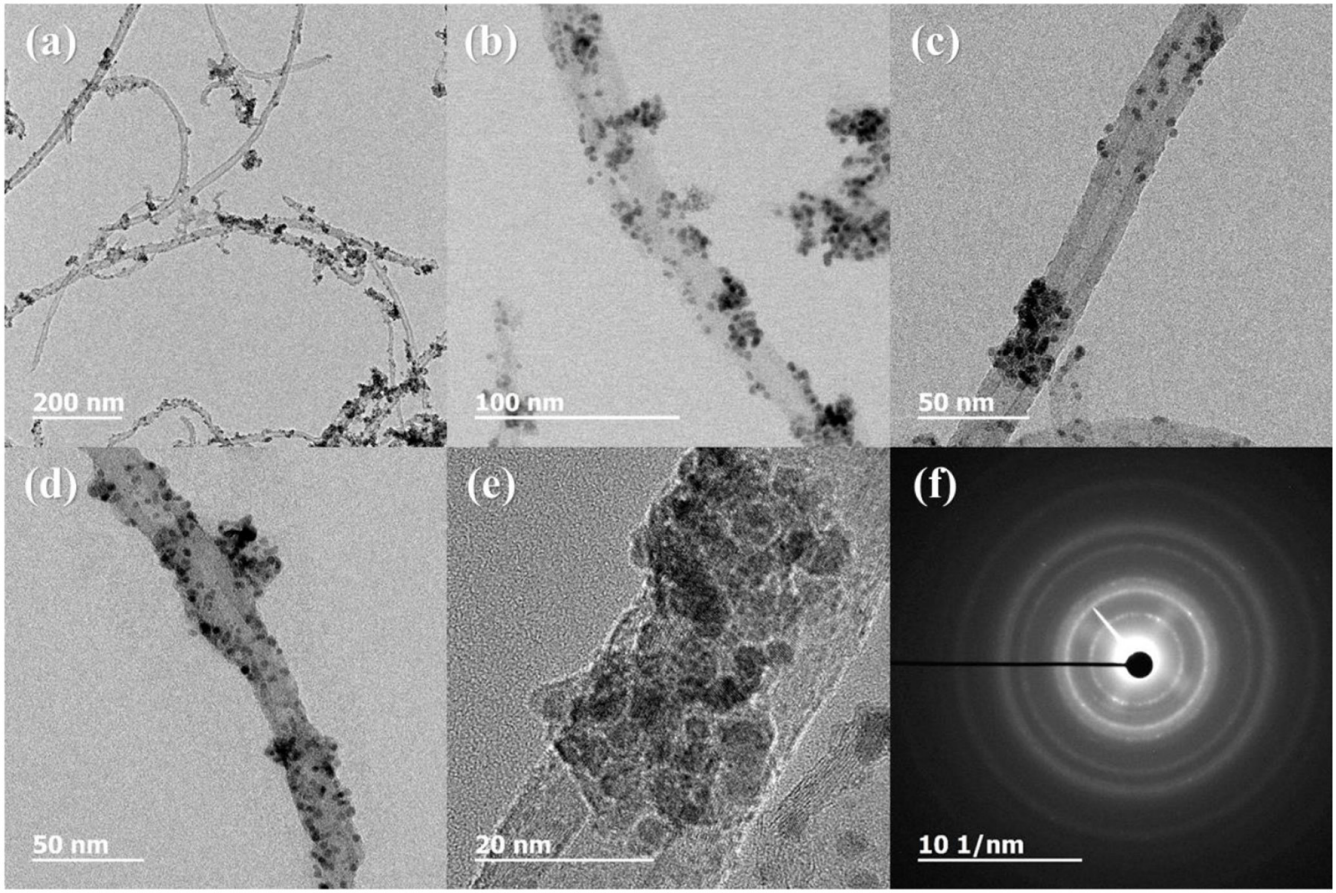
TEM and HR-TEM images and selected area electron diffraction (SAED) pattern of Ni-Pd/MWCNT. **(A)** 200 nm scale, **(B)** 100 nm scale, **(C,D)** 50 nm scale, **(E)** 20 nm scale, **(F)** diffraction pattern of Ni-Pd/MWCNT.

**Figure 5:**
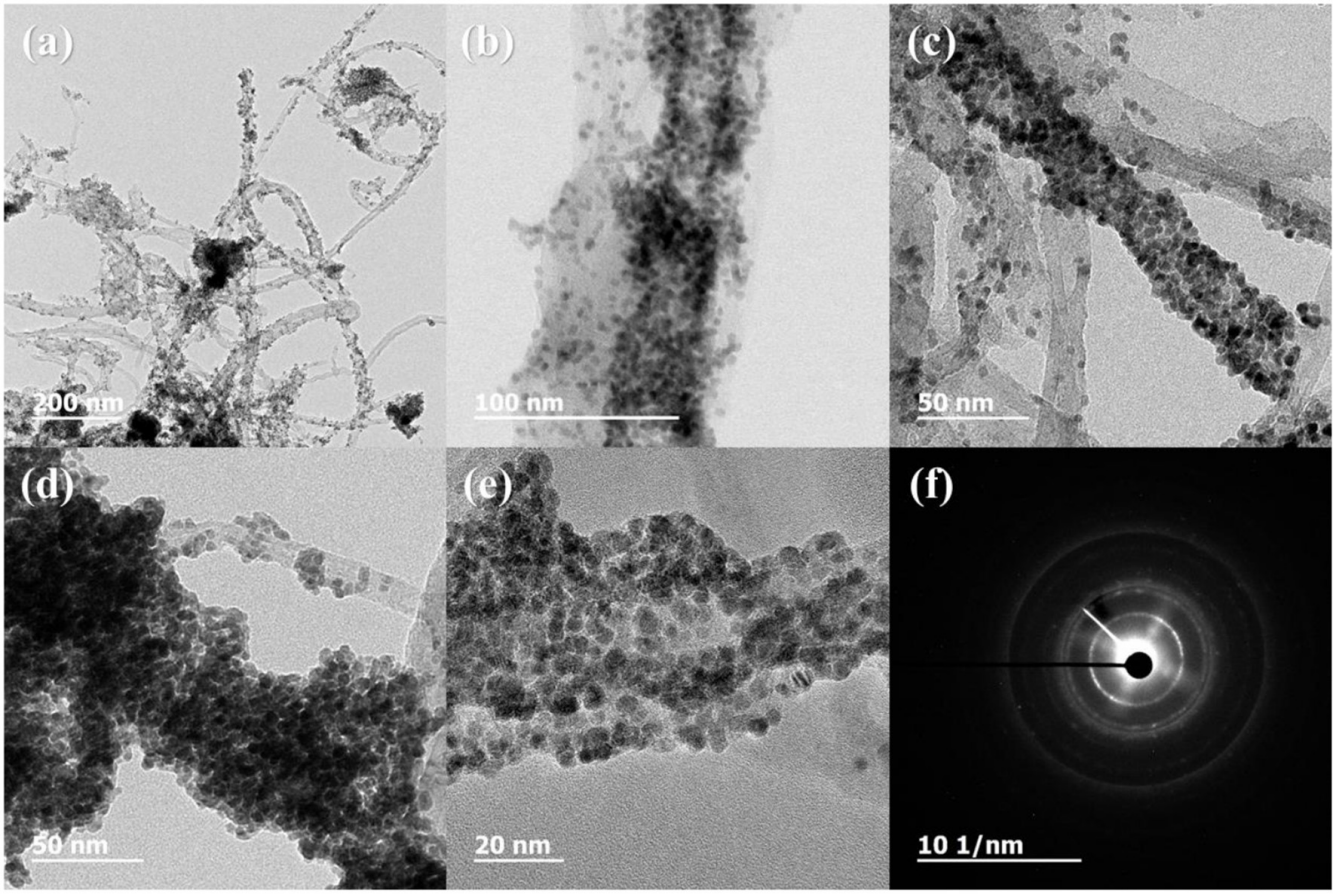
TEM and HR-TEM images and selected area electron diffraction (SAED) pattern of Ni-Pd(P)/MWCNT. **(A)** 200 nm scale, **(B)** 100 nm scale, **(C,D)** 50 nm scale, **(E)** 20 nm scale, **(F)** diffraction pattern of Ni-Pd(P)/MWCNT.

**Figure 6:**
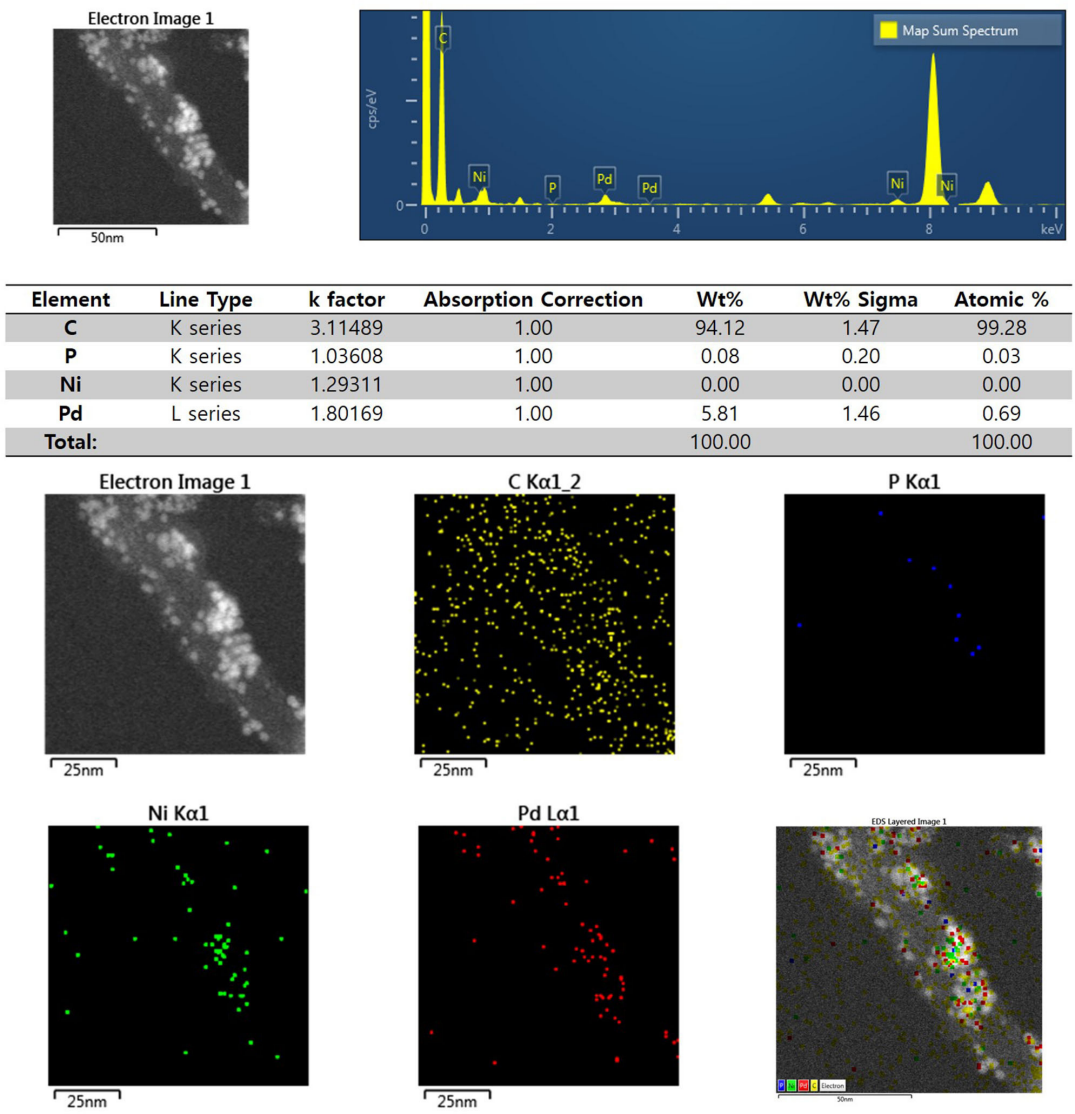
EDS elemental analysis results and elemental mapping images of Ni-Pd/MWCNT catalyst.

**Figure 7:**
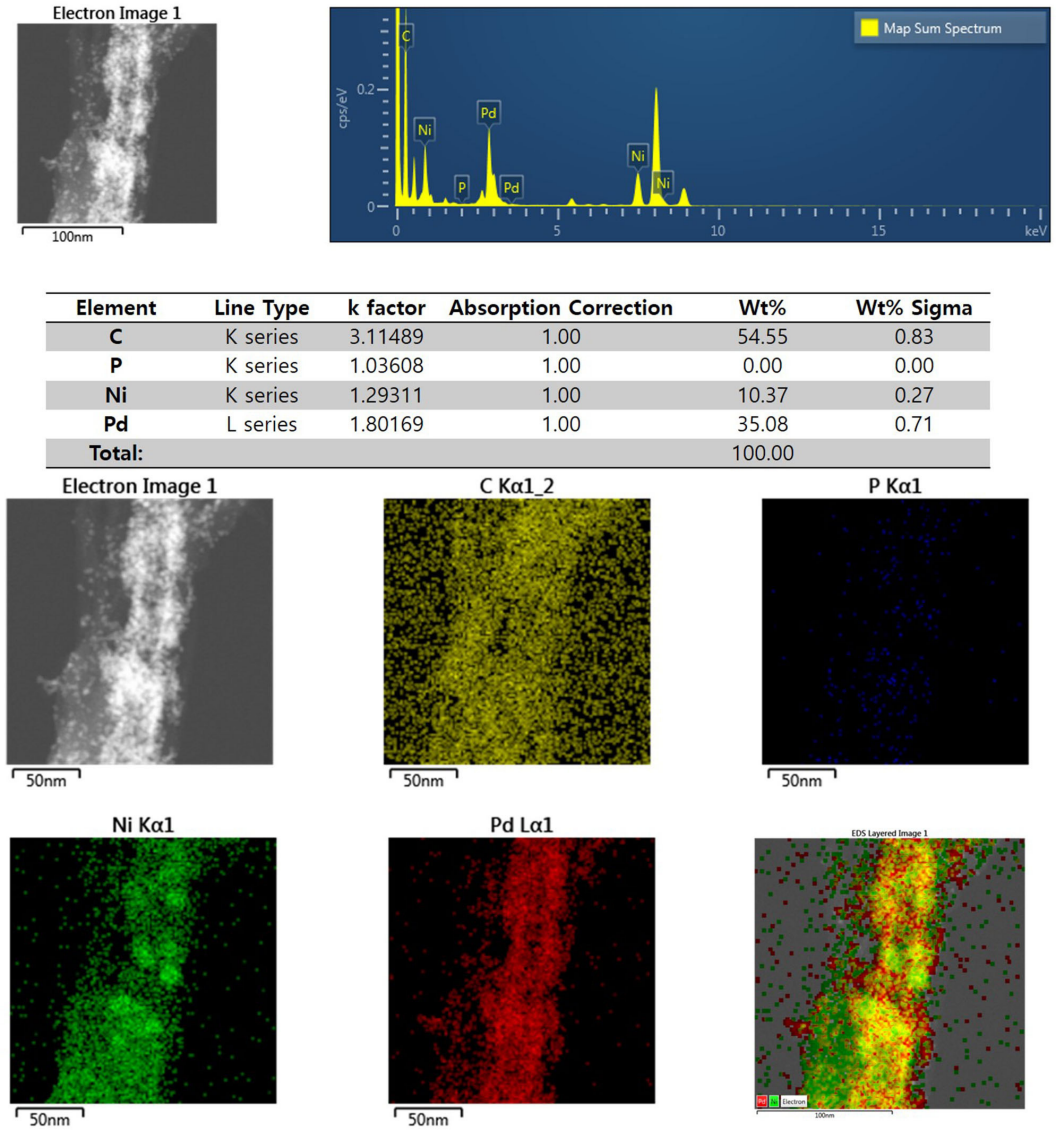
EDS elemental analysis results and elemental mapping images of Ni-Pd(P)/MWCNT catalyst.

Figure 3 shows the XRD profiles of Ni-Pd/MWCNT and Ni-Pd(P)/MWCNT catalysts. The XRD profiles show a diffraction peak at 25.8° corresponding to the (002) plane of carbon in MWCNTs. The Ni-Pd heteropoly alloy showed major peaks at 41.0° [PdNi(200)], 69.2°[PdNi(220)], and 82.0°[PdNi(331)] corresponding to d-spacings of 2.20 of PdNi (200) plane, 1.52 of PdNi (220) plane, and 1.17 of PdNi (331) plane, respectively. In the case of Ni-Pd(P)/MWCNT catalyst containing P [JCPDS No. 65-2491, P (Phosphorous)] (Yang et al., 2017), the intensities of the characteristic peaks of Pd at 31.2° and 46.7° [JCPDS No. 46-1043, Pd (Palladium)], respectively, decreased and a peak corresponding to the Ni-Pd alloy catalyst appeared at 41.0°. As a result of P (Phosphous) XRD peak, bimetallic crystal difference was rare between Ni-Pd, Pd and Ni [JCPDS No. 65-2865, Ni (Nickel)]. The catalyst can be checked for detailed surface properties through XPS characterization by further analysis (Wannasiri et al., 2020). This allows the monomeric metal catalysts of Ni and Pd formed at MWCNTs to be more dispersed over the catalytic support and thus identify the peak of the Ni-Pd catalytic structure, 41.0°, which is combined in a hetero-dispersive system (Yan et al., 2012; Liu et al., 2017). Compared with the catalyst without P, the P-containing catalyst exhibited a lower metal catalyst ratio of the monomeric system, which indicates that it is more hetero-deterministic. NaPO_2_H_2_.H_2_O acts as a reducing agent is in the pH 10 environment to help with the return of metallic salt with hydrazine, and to reduce particle size (Maiti et al., 2012). The peak at 41.0° in the XRD profile originates from the presence of P that remains on the MWCNT surface after the reduction of metal precursors to metal catalyst; however, it does not directly affect the catalyst characteristics. The average particle size is 5 nm, and the catalyst particles are distributed on the MWCNT surface. The diffraction peak at 25.8° corresponding to the [C (002)] plane of Ni-Pd in the Ni-Pd/MWCNT isomeric metal catalyst gives a crystallite size of 47Å. TEM and HR-TEM (**Figures 5**, **6**) analyses were performed to examine the structure on the surface. In the case of Ni-Pd/MWCNT in **Figure 5**, the catalysts produced on MWCNTs are located in the form of a lack of inter-electromagnetic manpower that is not combined with the actuators of the single-component catalysts and MWCNTs. Because of the uneven distribution of catalyst particles on the catalyst support, fuel oxidation does not occur (Wang et al., 2014; Ding et al., 2015; Periyasamy et al., 2016). After the addition of P by **Figure 6**, which decreased the catalyst particle agglomeration and changed the structural properties of the Ni-Pd catalyst, the catalyst particles were distributed more evenly on the catalyst support (Hur et al., 2020). This confirms that P addition led to the synthesis of a highly efficient Ni-Pd catalyst on the surface of the MWCNT catalyst support with enhanced fuel oxidation efficiency. The selected area electron diffraction (SAED) patterns of the Ni-Pd/MWCNT and Ni-Pd(P)/MWCNT catalyst particles are shown in Figures 4, 5, respectively. The SAED patterns of the catalysts confirm the polycrystalline nature of the Ni-Pd alloy. The crystal structure identified in SAED can identify the visible Ni-Pd grid plane (200) in the XRD pattern. Alloy catalyst Ni-Pd can check the distance between catalysts of 41.8°, 0.22 nm (SP.I. 1). The EDS analysis results of Ni-Pd/MWCNT shown in Figure 6 reveal the presence of C, O, Ni, and Pd as the main elements, whereas in the case of Ni-Pd(P)/MWCNT containing P, the intensity of Ni and Pd peaks are higher (Figure 7). This allowed comparison of synthesized Ni-Pd/MWCNTs with P added Ni-Pd(P)/MWCNTs by Figure 7, and the combination of metal alloys in the actuator produced by MWCNTs due to the dispersion and reduction of size and changes in structure characteristics of P (Basumatary et al., 2018), through XRD, TEM, and EDX.

### Electrocatalytic Performance for Urea Electro-Oxidation

**Figure 8:**
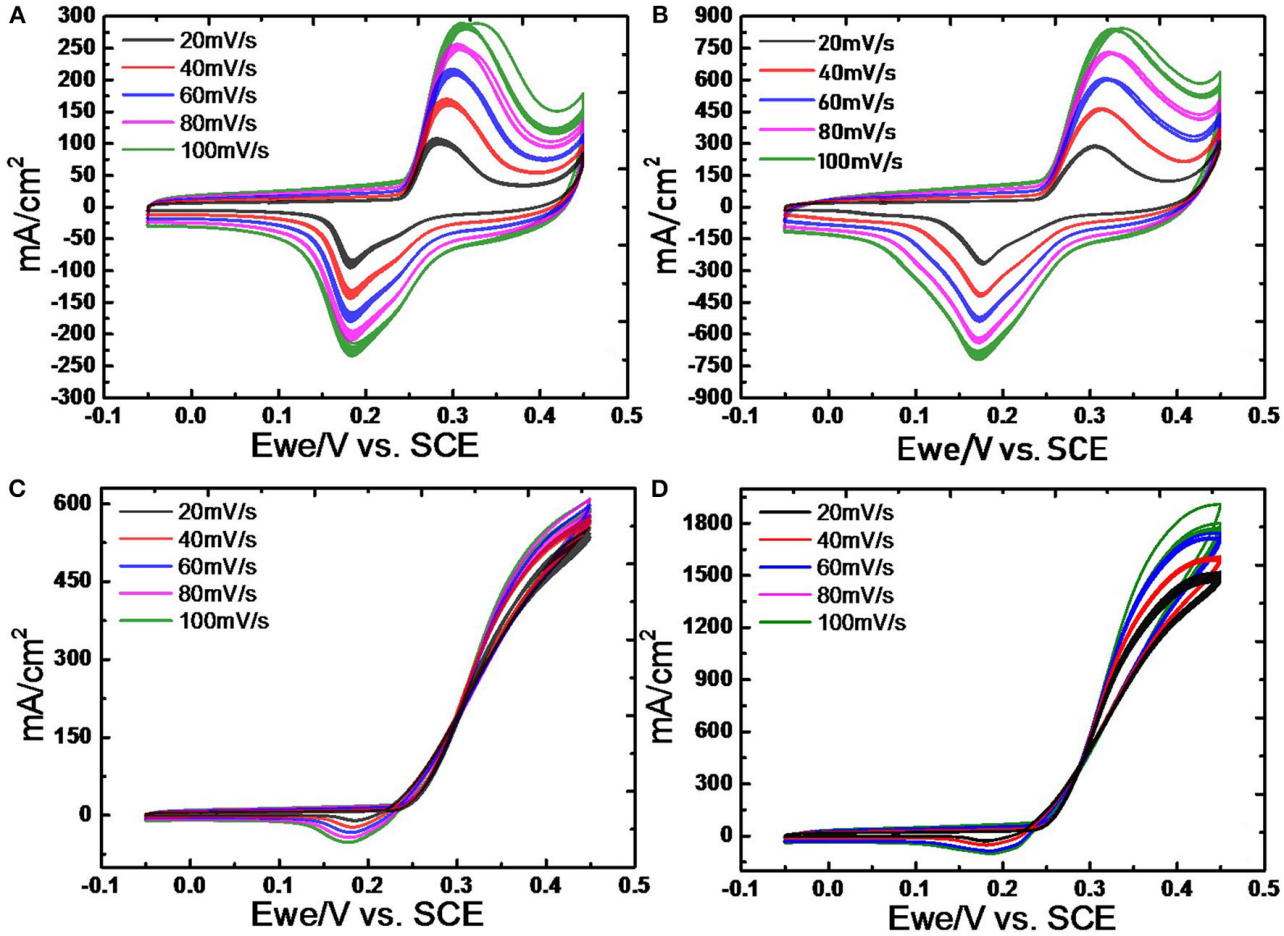
CV profiles of **(A)** Ni-Pd/MWCNT and **(B)** Ni-Pd(P)/MWCNT at scan rates of 20, 40, 60, 80, and 100 mV/s in 3.0 M KOH solution and 3.0 M KOH/1.0 M urea solution **(C)** Ni-Pd/MWCNT and **(D)** Ni-Pd(P)/MWCNT 20–100 mV/s scan rate.

Figure 8 shows the CV profiles of Ni-Pd(P)/MWCNT based on the linear injection potential method. CV measurements were carried out in 3.0 M KOH solutions at scan rates of 20, 40, 60, 80, and 100 mV/s. Based on the electrochemical reaction rate theory, in the case of catalyst-fuel reaction at a high reaction rate in electrochemical reaction, the rate at which ions spread is faster than the catalytic-fuel decomposition reaction rate; hence, the increase in current is limited. Therefore, the maximum CV scan rate was set as 100 mV/s. The reaction of catalyst with OH^−^ resulted in Ni(OH)_2_/NiOOH redox reaction. As shown in the figure, the onset potential decreased to 0.25 V due to the oxidation of urea, which was originally produced at 0.4 V (vs. Ag/AgCl). The current for oxidation increased with an increase in scan rate, and more electromagnetic activity occurred with the positive value (Woo et al., 2020). Ni-based catalysts react with urea fuel by oxidizing the metal catalyst Ni to Ni(OH)_2_, reducing to NiOOH to conduct fuel interaction, and γ-NiOOH at around 0.4 V (Lee et al., 2013a). In this electrochemical reaction, the fuel-catalyst was confirmed by the catalytic reaction leading from the onset to the Ni^2+^ reaction and to the Ni^3+^ reaction. This decelerates the reaction between the catalyst and fuel, and it adheres to the catalyst surface, thereby deactivating the catalyst. Thus, an Ni-Pd isomeric catalyst was used to prevent the occurrence of an overpotential where the reaction occurred at a higher location by making a response near 0.35 V. Figures 8A,B show the CV profiles of Ni-Pd/MWCNT and Ni-Pd(P)/MWCNT measured in a 3.0 M KOH solution. To confirm the excellent catalytic properties for urea oxidation, CV was performed at a scan rate of 20 mV/s, which is the rate at which sufficient reaction occurs. The peak current densities delivered by the catalysts at a low onset potential of 0.25 V were compared. Figures 8C,D show the CV profiles of Ni-Pd(P)/MWCNT measured in a 3.0 M KOH/1.0 M urea solution. During the reduction reaction of fuel at 0.23 V, the catalyst delivered a current density similar to that obtained for the oxidation reaction. At 0.45 V, the

**Figure 9:**
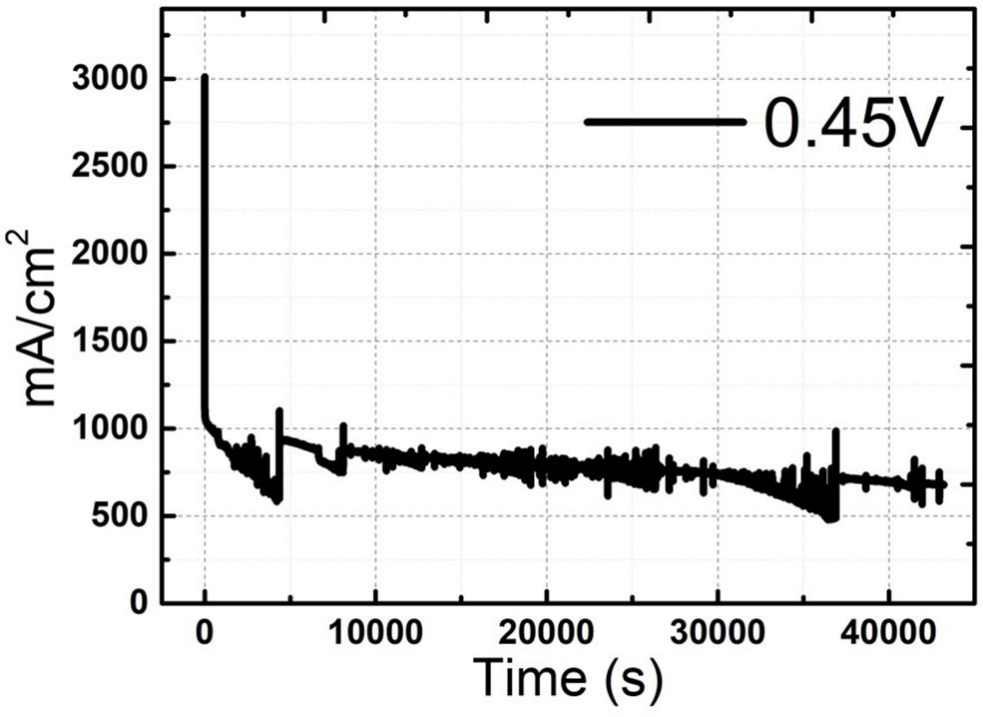
Chronoamperometric curve of Ni-Pd(P)/MWCNT in 3.0 M KOH/1.0 M urea solution after CV at 0.45 V for 40,000 s.

**Figure 10:**
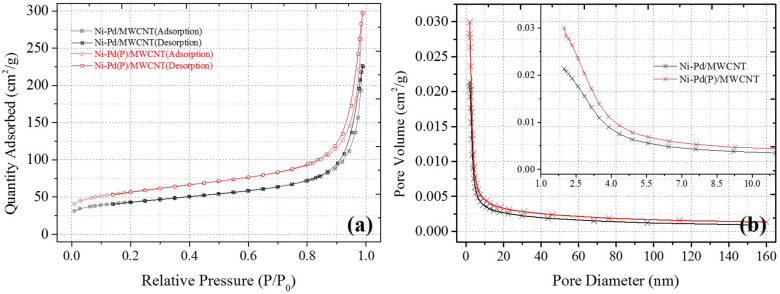
BET analysis results of Ni-Pd/MWCNT and Ni-Pd(P)/MWCNT: **(A)** Adsorption isotherms and **(B)** pore volume plots obtained after equilibrating for 10 s in N_2_ at 150°C.

Ni-Pd/MWCNT catalyst delivered a peak current density of 604.87 mA/cm^2^ (without P), whereas the Ni-Pd(P)/MWCNT catalyst delivered a current density of 1897.76 mA/cm^2^ (with P). Thus, the current density delivered by the P-containing catalyst was 3.13 times higher than that delivered by the catalyst without P. Ni-Pd(P)/MWCNT can confirm that the speed of the fuel-catalyst reaction, which occurs reversibly in the element oxidation reaction, is faster than the reaction rate of the catalyst without the addition of P. After the CV test in KOH without and with a urea solution, chronoamperometry was performed in an alkaline solution under 10% degradation for 15,000 s during CA of Figure 9, test 8 h, after finally noise in KOH alkaline solution at 0.45 V maximum current occur voltage. The even dispersion of Figure 10 the catalyst has increased the responsiveness, and the onset potential shows a constant but high current density, which implies an increase in the amount of catalyst participating in the reaction. Figure 10A shows that the BET (Brunauer–Emmett–Teller) surface areas of Ni-Pd/MWCNT and Ni-Pd(P)/MWCNT are 148.10 and 195.20 m^2^/g, respectively. The adsorption/desorption performance of the P-containing Ni-Pd(P)/MWCNT catalyst increased by 1.31 times over that of the Ni-Pd/MWCNT catalyst. Without P catalyst, reduction grain and pore size less than P added catalyst, Ni-Pd(P)/MWCNTs ~2.5 nm size of catalyst 1.4 times rich particle reduction reaction, as can be seen in Figure 10B. Based on the response speed, it is possible to determine the increased current density at which the element decomposition reaction occurs effectively, making the ion exchange suitable and synthesized under optimal conditions (Cha et al., 2018).

### Unit Cell Analysis of Direct Urea Fuel Cell

**Figure 11:**
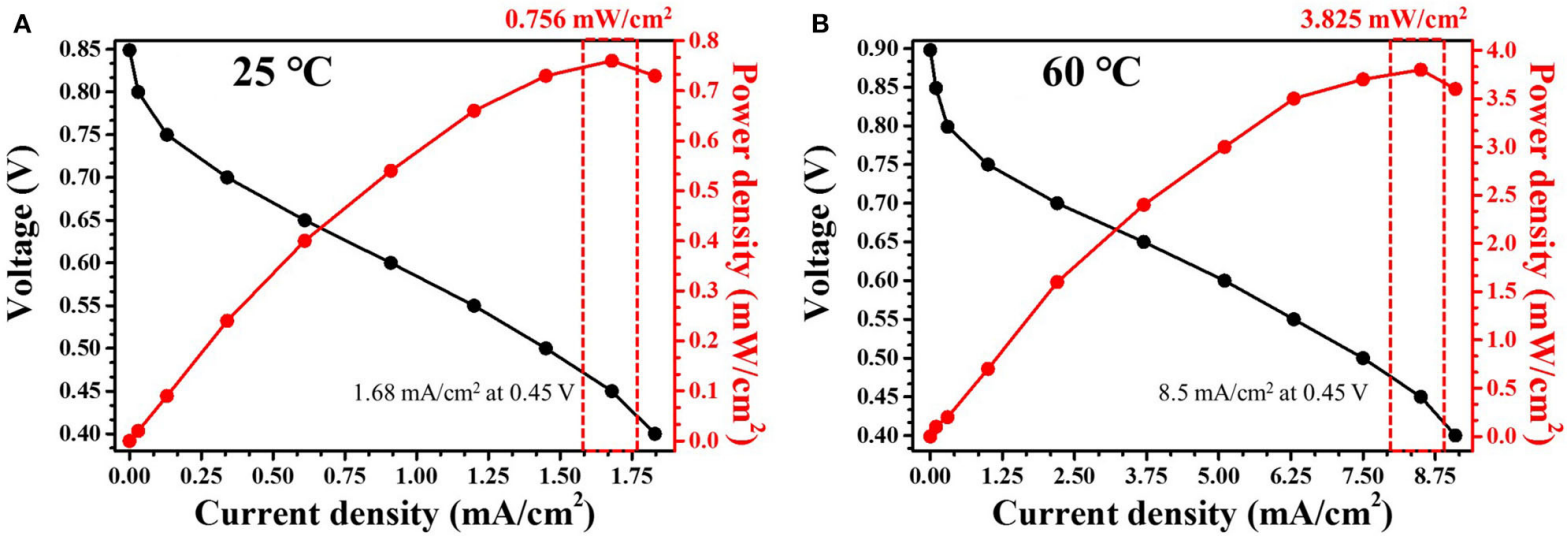
I-V curves obtained using Ni-Pd(P)/MWCNT catalyst in a 5 cm^2^ unit cell at **(A)** 25°C and **(B)** 60°C in 3.0 M KOH/1.0 M urea solution.

The optimum conditions for each solution concentration were examined, and the power density was determined for the Ni-Pd(P)/MWCNT catalyst. Conventional direct fuel cells have very low efficiencies and many drawbacks due to defects in the overvoltage and ion movement channels in the catalysts based on as much as applicable. Figure 11 shows the I-V curve of the fuel cell using Ni-Pd(P)/MWCNT. At 25°C, the OCV was 0.86 V, which is ~75% of the theoretical value [Eq.1 (Overall votage, 1.146 V)], and the maximum output was 0.756 mW/cm^2^ at 0.45 V. At 60°C, the OCV was 0.90 V, which is ~78% of the theoretical value, and the maximum output was 3.825 mW/cm^2^, which is ~5 times higher than that measured at room temperature. The decomposition of the element is formed and decomposed into Ni(OH)_2_, NiOOH when encountered with Ni in a base environment. At this point, a chain reaction begins as the main response of NiOOH, which requires higher energy, stabilizes to Ni(OH)_2_. The optimal conditions are a pH of 12–13 and a temperature of 60°C, and the optimal Ni hydroxide compounds can be identified. The above temperature conditions are based on the application of an anion polymer separator. Although it is the result of the experiment based on the electrochemical test, only the differential is confirmed, but an increase in linear current density is not observed.

## Discussion

Ni-Pd(P)/MWCNT as a high-efficiency catalyst mitigated the overpotential problem associated with the use of Ni monometallic catalysts with a low onset potential and major response voltage due to the addition of Pd metal. Moreover, the addition of P, which was used as a dispersant to resolve the problem of surface control of the Ni-Pd alloy catalyst, led to the uniform distribution of the catalyst on the catalyst support through the reduction in size and surface area control. The hydrothermal synthesis method employed in this study is simple and would enable the mass production of the catalyst. The catalyst gave rise to a strong urea oxidation peak and delivered an excellent peak current density depending on voltage. Ni-Pd/MWCNT and Ni-Pd(P)/MWCNT delivered current densities of 285.88 (without P) and 838.05 mA/cm^2^ (with P) at 0.32 V (vs. Ag/AgCl) in a 3.0 M KOH solution and 604.87 mA/cm^2^ (without P) and 1897.76 mA/cm^2^ (with P)at 0.45 V (vs. Ag/AgCl) in a 3.0 M KOH/1.0 M urea solution. Based on the CV results, the optimal 3.0 M KOH/1.0 M urea alkaline aqueous solution was used in a 5 cm^2^ unit cell. Ni-Pd(P)/MWCNT containing P is a promising anode catalyst for a DUFC and delivered ~5.0 times higher current density (3.825 mW/cm^2^) compared with the catalyst without P (0.756 mW/cm^2^) at 25°C. The maximum current densities at 25 and 60°C were also measured at 0.45 V. Nevertheless, improving the power density of DUFCs would require not only high-efficiency catalysts, but also resolving other problems in DUFCs. However, this catalyst can be used to improve the current density that can considerably contribute toward the commercialization of DUFCs. Therefore, Ni-Pd(P)/MWCNT is a promising anode catalyst for DUFCs.

## Data Availability Statement

All datasets presented in this study are included in the article/supplementary material.

## Author Contributions

UL, YL, and YY listed have made a substantial, direct and intellectual contribution to the work, and approved it for publication. All authors contributed to manuscript revision, read, and approved the submitted version.

## Conflict of Interest

The authors declare that the research was conducted in the absence of any commercial or financial relationships that could be construed as a potential conflict of interest.
