# Corrigendum: Enhanced electrochemical properties of catalyst by phosphorous addition for direct urea fuel cell

**DOI:** 10.3389/fchem.2024.1400748

**Published:** 2024-04-02

**Authors:** Unho Lee, You Na Lee, Young Soo Yoon

**Affiliations:** Materials Science and Engineering, Gachon University, Seongnam-si, Republic of Korea

**Keywords:** direct urea fuel cell, urea, phosphorous addition, anode catalyst, anion exchange fuel cell, hydrothermal synthesis, Ni-Pd alloy, MWCNTs

In the published article, there was an error in the **Funding** statement. Gachon University was omitted from the statement. The correct statement is as follows:

“This work was supported by the National Research Foundation of Korea (NRF) grant funded by the Korea government (MSIT) (No. NRF-2019M2D1A1079208), and this work was supported by the Gachon University research fund of 2020(GCU-202008470008).”

The authors apologize for this error and state that this does not change the scientific conclusions of the article in any way. The original article has been updated.

